# Re-wiring and gene expression changes of AC025034.1 and ATP2B1 play complex roles in early-to-late breast cancer progression

**DOI:** 10.1186/s12863-021-01015-9

**Published:** 2022-01-14

**Authors:** Samane Khoshbakht, Majid Mokhtari, Sayyed Sajjad Moravveji, Sadegh Azimzadeh Jamalkandi, Ali Masoudi-Nejad

**Affiliations:** 1 Laboratory of Systems Biology and Bioinformatics (LBB), Department of Bioinformatics, Kish International Campus, University of Tehran, Kish Island, Iran; 2 Chemical Injuries Research Center, Systems Biology and Poisonings Institute, Tehran, Iran; 3 Laboratory of Systems Biology and Bioinformatics (LBB), Institute of Biochemistry and Biophysics, University of Tehran, Tehran, Iran

**Keywords:** Prognostic biomarker, ER-positive breast cancer, Differential network, Stage, Systems biology, Re-wiring, Dynamic changes

## Abstract

**Background:**

Elucidating the dynamic topological changes across different stages of breast cancer, called stage re-wiring, could lead to identifying key latent regulatory signatures involved in cancer progression. Such dynamic regulators and their functions are mostly unknown. Here, we reconstructed differential co-expression networks for four stages of breast cancer to assess the dynamic patterns of cancer progression. A new computational approach was applied to identify stage-specific subnetworks for each stage. Next, prognostic traits of genes and the efficiency of stage-related groups were evaluated and validated, using the Log-Rank test, SVM classifier, and sample clustering. Furthermore, by conducting the stepwise VIF-feature selection method, a Cox-PH model was developed to predict patients’ risk. Finally, the re-wiring network for prognostic signatures was reconstructed and assessed across stages to detect gain/loss, positive/negative interactions as well as rewired-hub nodes contributing to dynamic cancer progression.

**Results:**

After having implemented our new approach, we could identify four stage-specific core biological pathways. We could also detect an essential non-coding RNA, *AC025034.1*, which is not the only antisense to *ATP2B1* (cell proliferation regulator), but also revealed a statistically significant stage-descending pattern; Moreover, *AC025034.1* revealed both a dynamic topological pattern across stages and prognostic trait. We also identified a high-performance Overall-Survival-Risk model, including 12 re-wired genes to predict patients’ risk (c-index = 0.89). Finally, breast cancer-specific prognostic biomarkers of *LINC01612*, *AC092142.1*, and *AC008969.1* were identified.

**Conclusions:**

In summary new scoring method highlighted stage-specific core pathways for early-to-late progressions. Moreover, detecting the significant re-wired hub nodes indicated stage-associated traits, which reflects the importance of such regulators from different perspectives.

**Supplementary Information:**

The online version contains supplementary material available at 10.1186/s12863-021-01015-9.

## Background

Breast cancer is one of the most prevalent cancers among women all around the world. According to the World Health Organization (WHO) reports in 2018, it includes a high-frequency cancer rate, [1]. To take more appropriate treatments in the clinic for breast cancer patients, several computational/non-computational studies have been conducted to improve prognostic staging systems through assessment of biomarkers, including estrogen receptor status (ER) and human epidermal growth factor receptor 2 status (HER2) for breast cancer patients, or using the predictive recurrence models, such as Oncotype DX [2–4]. Therefore, the surveys on detecting novel prognostic biomarkers, including protein-coding (PC) /non-coding (NC) RNAs relating to cancer dynamics across stages, would be of great interest for more precise therapeutic decisions, as well as avoiding metastasis in breast cancer [3, 5, 6].

Multiple predisposing and triggering factors are involved in cancer progression, including genetics, epigenetics, and environmental driver events [7, 8]. Such hidden events adversely affect gene expression or gene regulatory associations, contributing to mechanistic molecular/cellular disorders [9]. Negative loss/gained functions of genes or changes among gene expression interactions (co-expression re-wiring) in biological networks could propagate and develop advanced cancer stages [10, 11]. In the case of cancer complexities, dysregulated pathways including DNA damages leading to Epithelial-Mesenchymal Transition (EMT), cell proliferation, morphogenesis, as well as dissemination of tumor cells can emerge during different breast cancer stages [12–14]. Therefore, the implementation of the systems biology approaches on cancer studies for a better perceiving of such complexities is promising [15, 16]. Among different approaches, differential co-expression analysis can be employed for the identification of the involved key gene signatures that may not be detectable through differential expression analyses or co-expression analyses [9, 17–19]. In which, characterization of re-wired subnetworks can reveal the reprogramming of gene expression regulations across different disease conditions [6, 9, 20]. Therefore, using assessing re-wiring topological traits through systems biology approaches would result in understanding latent biological insights of breast cancer.

In the present study, we focused on the comprehensive assessment of dynamic modular variations, re-wiring, among gene interactions resulting from cancer progression in estrogen-receptor-positive (ER+) breast cancer patients (315 patients included). We identified four stage-specific subnetworks which revealed core pathways for each stage of breast cancer. The stage- and breast cancer-specificity of subnetworks were assessed through a new computational approach. To identify breast cancer-specific prognostic biomarkers, we implemented the Log-Rank test and Kaplan-Meier curve for breast cancer, as well as other 32 TCGA cancer types. We could detect stage-associated gene signatures, applying the Kruskal-Wallis and Post-Hoc tests. Furthermore, we applied the VIF-feature selection method to identify an Overall-survival-risk model consisting of a few genes to predict patients’ risk. Finally, co-expression networks were reconstructed for four stages of breast cancer, and the re-wiring among prognostic genes was assessed across stages. The gain, loss, and reverse interaction-hub nodes were detected across stages. The survival results were validated, using SVM classification, hierarchical clustering, Log-Rank test.

## Results

**Fig. 1:**
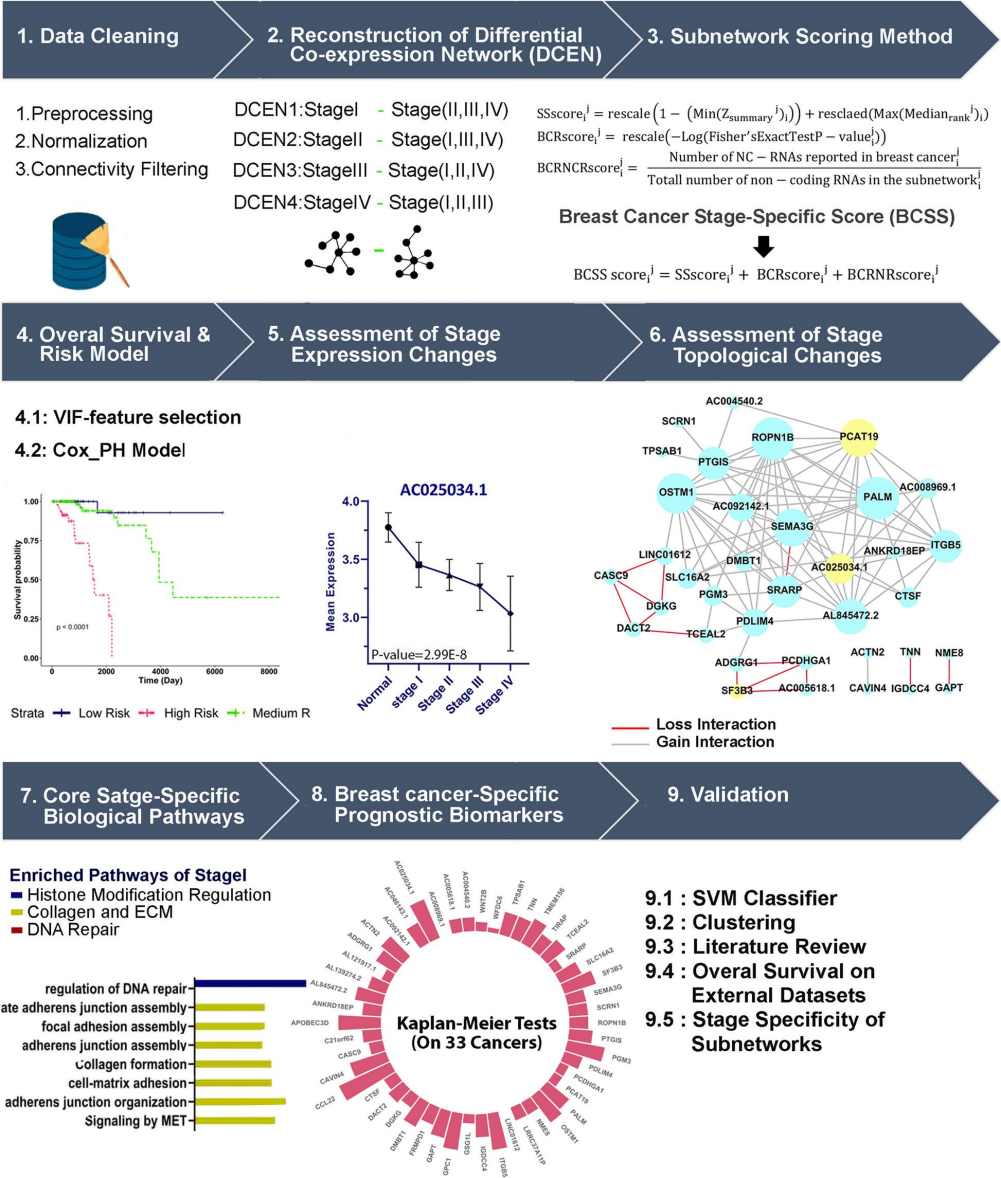
A comprehensive assessment of breast cancer progression outline. The first step is data cleaning and normalization, the second step is Differential co-expression network (DCRN) reconstruction for four stages, and the third step is the computational approach for scoring and extracting breast cancer-related stage-specific (BCSS) subnetworks for each stage. In the fourth step, the survival analyses were implemented for four BCSS subnetworks, and a risk model fitted to data. In step five, the stage-related genes were detected; in step six, the topological changes, called re-wiring, among prognostic genes were assessed across stages. In step seven, the core biological pathways for stage-specific subnetworks were detected; in step eight, the breast cancer-specific prognostic genes were detected, and finally in step nine, the computational validation were implemented

The outline of our study was illustrated in Fig. 1 (The supplementary material was provided in the Supplementary material file).

### Differential co-expression network (DCEN) reconstruction

After normalization and gene filtering, the DCENs for four stages of breast cancer were reconstructed based on stage grouping (Supplementary Table S1). Concerning the therapeutic importance of HER-2 status of ER-positive patients, we reconstructed the differential network between HER2 positive and negative and extracted subnetworks, but we did not detect any HER2-related subnetwork. Moreover, we assessed the difference between HER2 positive and HER2 negative employing t-test, PCA analysis, and hierarchical clustering. There was no significant difference between them (Supplementary Table S2, 3, Supplementary Fig. S1, S2). Finally, we also implemented the differential expression (DE) analysis between HER2 positive and HER2 negative and found merely one differentially expressed gene.

### Breast cancer related and stage-specific subnetworks

**Fig. 2:**
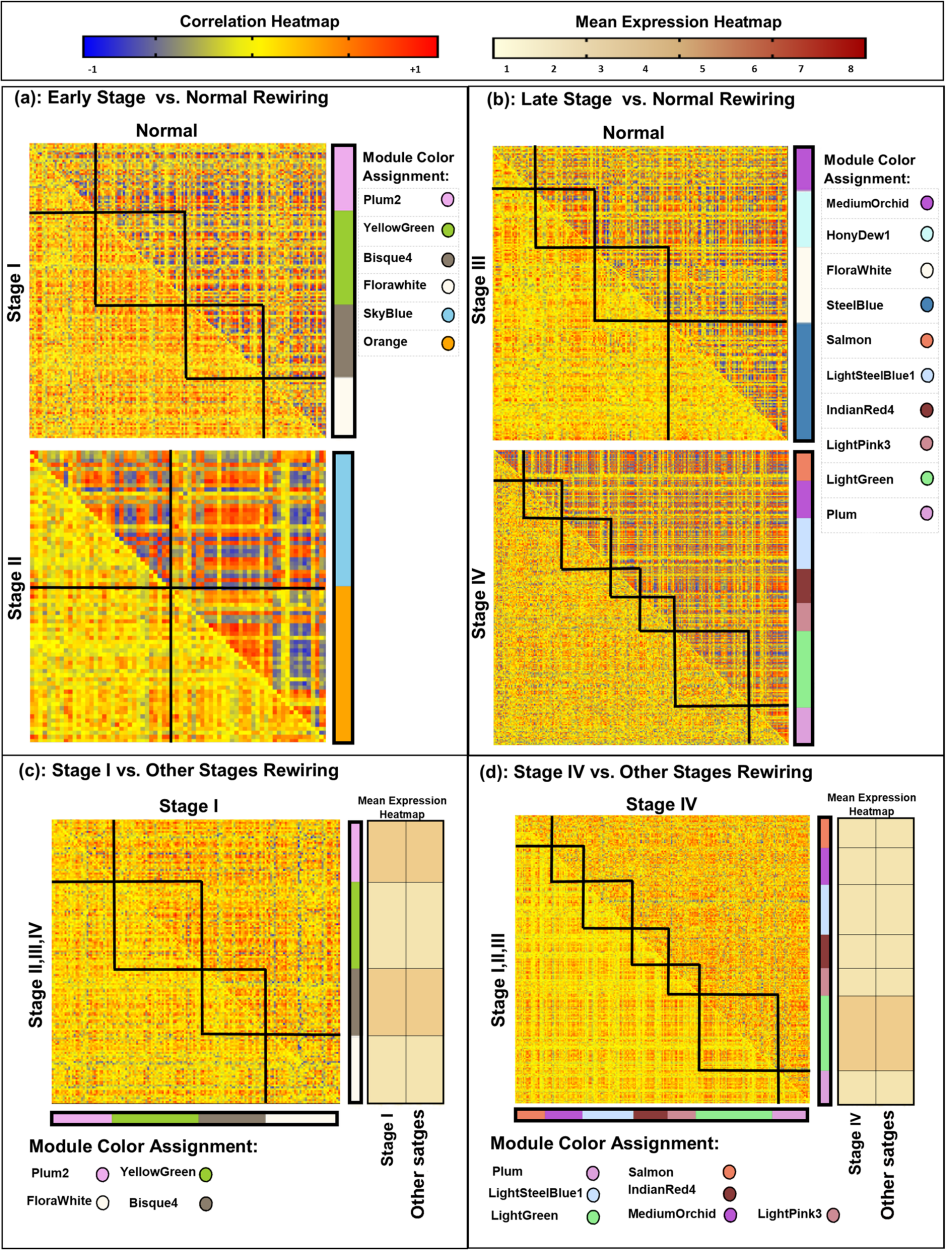
Overall re-wiring view of breast cancer-related subnetworks. For simplicity, each subnetwork was assigned by color and they were illustrated by circles. x and y axes for each heatmap show different stages or the normal condition. Each subnetwork was separated by a black line. And, the squares highlighted re-wiring changes between two conditions. In the re-wiring heatmaps, the red color indicates positive associations, the blue color indicates negative associations and the yellow color indicates weak associations among genes. In the mean expression heatmaps, the brown intensity indicates the mean expression of subnetworks

Hierarchical clustering was applied to DCENs for four stages to extract all re-wired subnetworks (Supplementary Fig. S3). The name of subnetworks was indicated by color. Most breast cancer-related and stage-specific subnetworks were detected for each stage, using the BreastCancerStageSpecific score (BCSS) scores (1< BCSS score_*i*_ < 4, *i* indicate stages) (Supplementary Table S4,S5,S6,S7). The overall re-wiring changes between every two conditions (four stages and normal tissue) were assessed (Fig. 2).

**Fig. 3:**
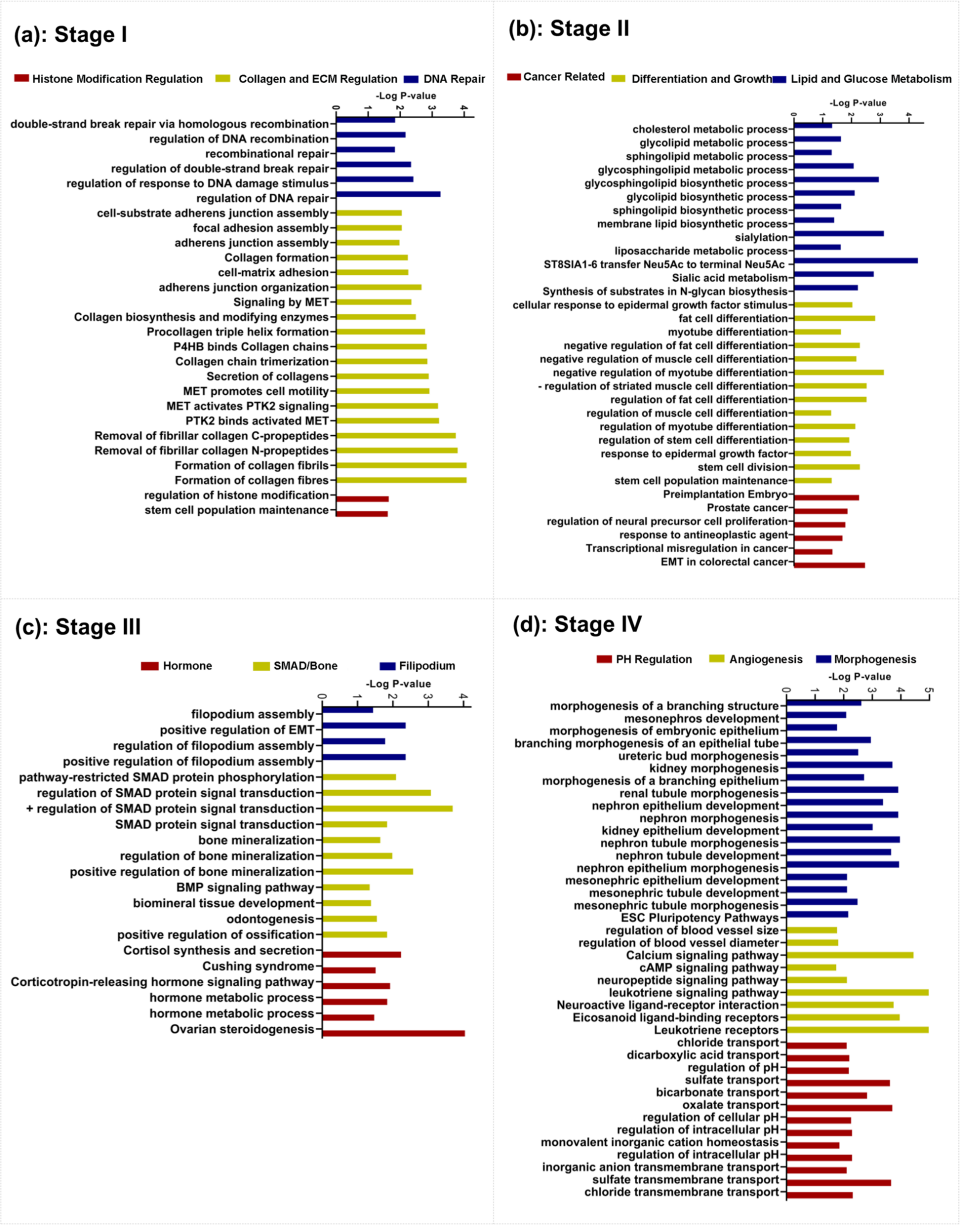
Functional geneset enrichment analysis of breast cancer-related stage-specific (BCRSS) subnetworks. Each color of bar charts represents a biological process term. The bar length indicates the minus log(*P*-value). **a**) indicates biological pathways of BCRSS subnetwork for stage (FloralWhite). **b**) indicates biological pathways of BCRSS subnetwork for stage II (Orange). **c**) indicates biological pathways of BCRSS subnetwork for stage III (FloralWhite2). **d**) indicates biological pathways of BCRSS subnetwork for stage IV (IndianRed4)

In Fig. 2, the circles indicate names of subnetworks and black squares indicate the re-wirings of genes belonging to a particular subnetwork across two conditions (x and y axes indicate a stage or normal condition). In the re-wiring heatmaps, the red color indicates the positive correlations, and the blue color indicates negative correlations among genes (Fig. 2,a,b,c,d). We also compared the gene expression change between two conditions, using mean expression heatmaps besides re-wiring heatmaps in Fig. 2c,d; In which, the brown color indicates gene expression intensity. The molecular interactions showed faded positive/negative associations or reversed ones in cancerous tissue in comparison to normal (Fig. 2a, b). While the mean expression of subnetworks did not reveal any change, the subnetworks of stage І (Bisque4, FloralWhite, Plum2, and YellowGreen) and stage IV (Plum, LightSteelBlue1, LightGreen, Salmon, IndianRed4, MediumOrchid, and LightPink3) showed the prominent re-wiring intensities compared to other stages, distinctly for stage IV (Fig. 2c, d). We could detect the highest BCSS scores for the FloralWhite in stage I (BCSSscore = 2.92), Orange in stage II (BCSSscore = 3.12), FloralWhite2 in stage III (BCSSscore = 2.69), and the Indianred4 in stage IV (BCSSscore =2.02) (Supplementary Table S6). Therefore, they indicated the selected breast cancer-related stage-specific (BCRSS) subnetworks for stages I, II, III, and IV, respectfully. BCRSS subnetworks were functionally enriched (Fig. 3).

We could detect ‘DNA Repair’, ‘Collagene/ECM Regulation’, and ‘Histone Modification’ pathways for stage I, ‘Lipid/Glucose Metabolism’, ‘Differentiation/Growth’, and ‘Cancer-Related’ pathways for stage II, ‘Filipodum’, ‘SMAD/Bone’, and ‘Hormone’ pathways for stage III, and ‘Morphogenesis’, ‘Angiogenesis’, and ‘PH-Regulation’ for stage IV (Fig. 3a, b, c, d).

### Overall survival (OS) analyses

**Fig. 4:**
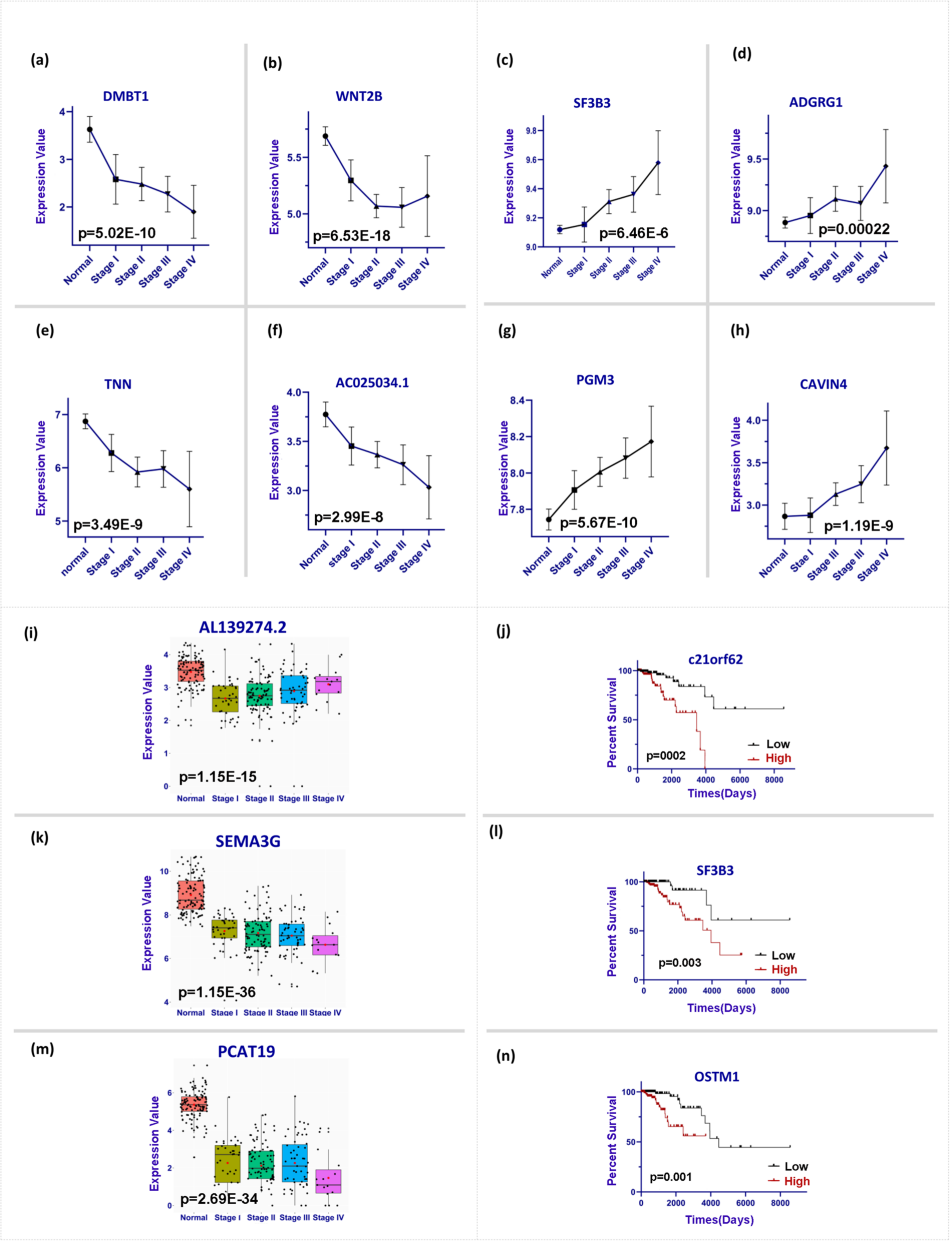
Stage-associated genes. The sections of a, b, e, and f indicate stage-descending prognostic genes (P indicates Kruskal-Wallis test p-value). The sections of c, d, g, and h indicate stage-ascending prognostic genes (P indicates the Kruskal-Wallis test P-values).  The sections of i, k, and m indicate box-plots for the outset-cancer group; The t-test P-values (P) were reported. the sections of j, l, and n indicate Kaplan-Meier curves and P-values for the Log-Rank test. Patients were separated by the median value of a gene. ‘Low’ indicates gene expression lower than the median and ‘High’ indicated the gene expression higher than the median. In all parts the significance level was 0.05

We could identify 50 prognostic genes including 19 genes (PC and NC) in stage I, four genes (PC) in stage II, 15 genes (PC and NC) in stage III, and 12 genes (PC and NC) in stage IV. The Kaplan-Meier curves and Log-rank *P*-values of *c21orf62*, *SF3B3*, and *OSTM1* were illustrated (Fig. 4j, l, n); In which, their high expressions were associated with the patient’s low survival rate. The expression trends of 50 prognostic genes (Supplementary Table S7) were classified into three groups of the stage-descending, stage-ascending, and outset-cancer group (Fig. 4i, k, m). The outset-cancer category showed a high expression level between normal and stage I (Fig. 4). Based on the literature review, 15 out of 50 genes are reported as prognostic genes in multiple studies on breast cancer (Supplementary Table S7). To detect the stage-associated genes, we implemented the Kruskal-Wallis and Post-Hoc tests on 50 genes, and we reached *SF3B3*, *ADGRG1*, *PGM3*, *SEMA3G*, *CAVIN4*, *AL139274.2*, and *PCAT19* which could fairly cluster the stage of samples (Supplementary Fig. S4).

#### Stage-rewiring networks

**Fig. 5:**
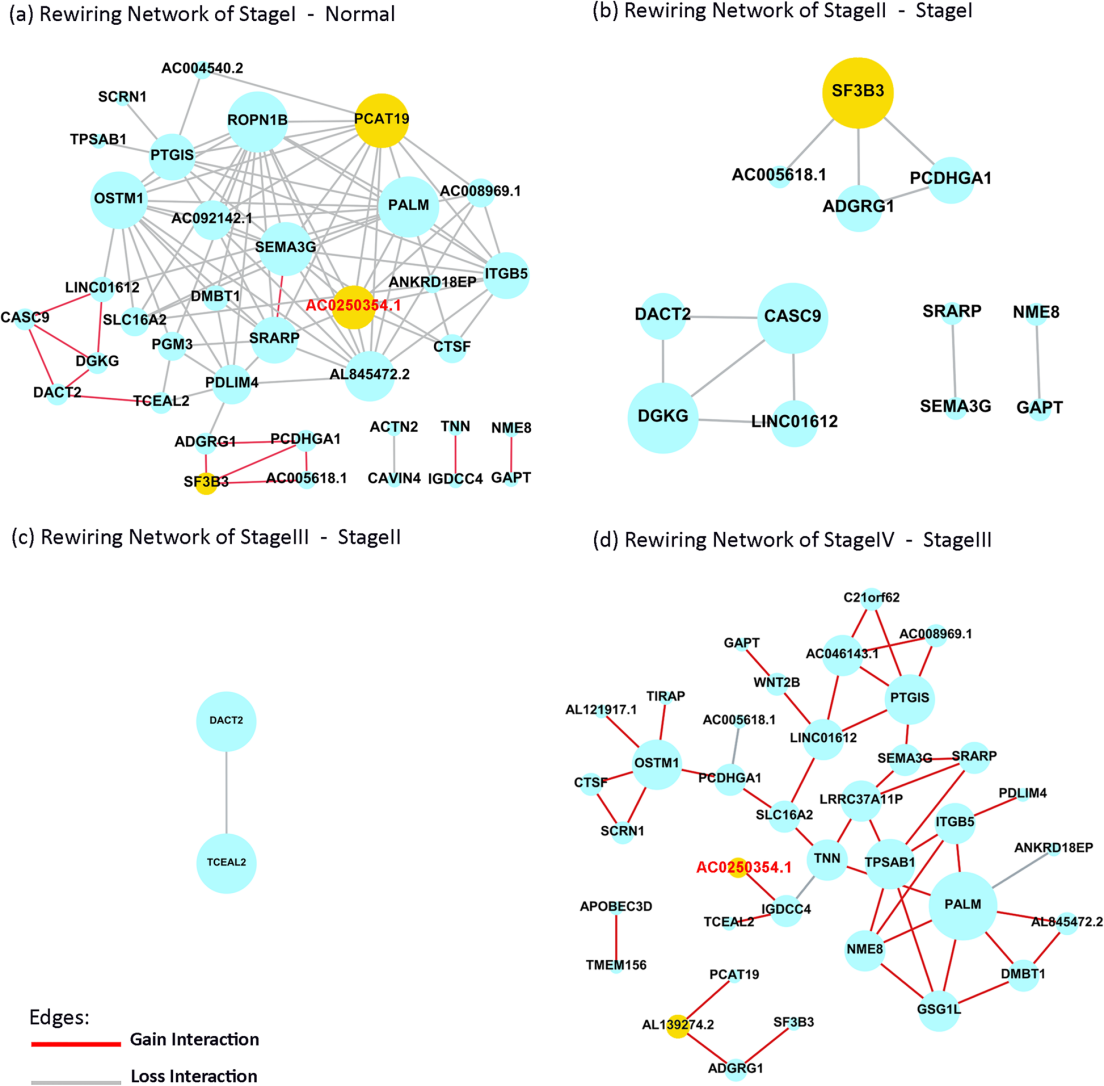
Stage-rewiring network. Red lines indicate gain interactions, grey lines indicate loss interactions, and the yellow nodes indicate important re-wired genes. a) re-wiring in stage I vs. normal condition. b) re-wiring in stage II vs. stage I. c) re-wiring in stage III vs. stage II. d) re-wiring in stage IV vs. stage III

The stage-rewiring networks of 50 prognostic genes were reconstructed (Fig. 5). Dynamic conditions (differential networks) included Stage I-Normal (co-expression in stage I minus co-expression in Normal), Stage II-Stage I, Stage III-Stage II, and Stage IV-Stage III (Fig.5 a, b, c, d). Stage I-Normal (gain = 14, loss = 83) and stage IV-stage III (gain = 45, loss = 3) differential networks showed more re-wiring among genes in contrast to stage II-stage I (loss = 11) and stage III-stage II (loss = 1) differential networks. Comparing stage I to normal, more interactions were lost (grey interactions). And, we could detect more gain interactions in stage IV vs. III (red interactions). Furthermore, we could identify loss-hub nodes in stage I and gain-hub nodes in stage IV (larger node size indicates hub nodes).

#### OS predictive model

**Fig. 6:**
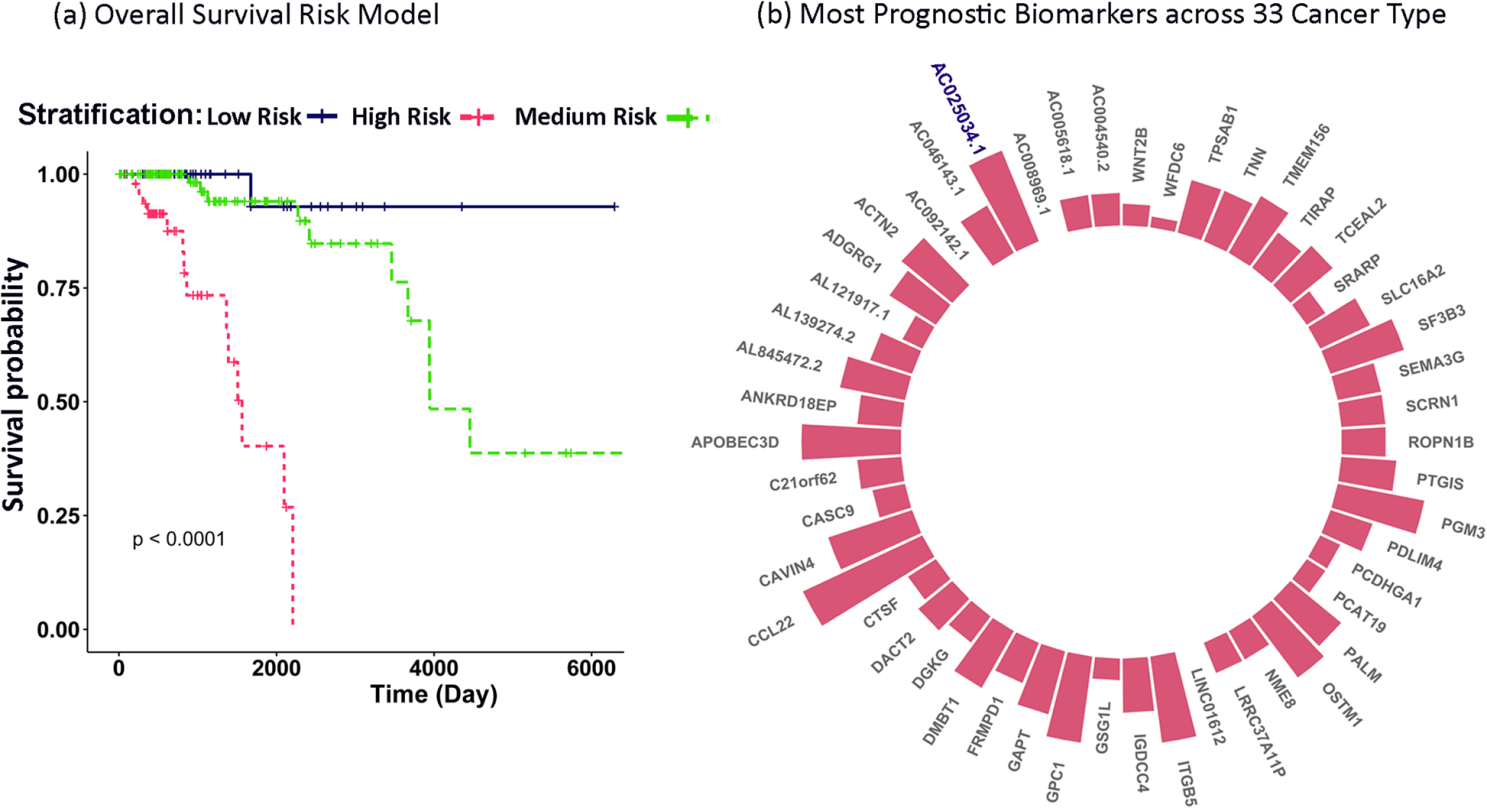
Overall-survival-risk model and prognostic genes in 33 TCCGA cancer types. a) Kaplan-Meier curve and Log-Rank *P*-value for the predictive Overall-survival-risk model, b) 33 TCGA cancers were assessed. The height of bar charts indicates the number of cancers that a gene was significantly prognostic for it

We identified 12 significant covariates in Overall-survival-risk model including *AC004540.2*, *GPC1*, *ACTN2*, *LINC01612*, *LRRC37A11P*, *SRARP*, *ADGRG1*, *PCAT19*, *ITGB5*, *GPC1*, *SEMA3G*, *SF3B3* (likelihood-ratio-test *P*-value = 3.764E-07). The identified model could precisely stratify patients into three groups of the low, medium, and high risk (Log-rank *p*-value = 0.00001) (Fig. 6a). The hazard ratio values of covariates and *p*-values were reported in Supplementary Table S7 and S8, in which the SF3B3 has the highest hazard ratio value (HR = 6.9), indicating its importance in patients’ low survival and also confirmation for ascending expression trend across stages (Fig. 4c,l). The concordance index (c-index), demonstrating the high performance of our survival model in obtaining patients’ risk scores, is 0.89.

#### Prognostic validation

The prognostic genes were validated by an external dataset and also in the Gepia web server (OS of 50 prognostic genes in 33 TCGA cancer types) (Supplementary Table S11). We further observed that 23 of our genes were validated in Kidney renal clear cell carcinoma (KIRC) and 18 genes in Brain Lower Grade Glioma (LGG), and 13 genes were validated in breast cancer (Supplementary Table S11). LncRNAs *LINC01612*, *AC092142.1*, and *AC008969.1* were prognostic just in breast cancer; Therefore, they may be nominated as breast cancer-specific prognostic non-coding biomarkers (Fig. 6b). We also validated the stage specificity of the final stage-specific subnetworks in the ER-negative group. For every stage, the *Z*_*summary*_ values were lower than 2, and the *Median*_*rank*_ values were high enough to conclude that subnetworks were stage-specific in ER-positive samples (Supplementary Table S4). We could not validate the specificity of the identified subnetworks in stage four of ER-negative data due to the very limited number of samples.

#### Clustering/classification

The outset-cancer validation was implemented using the SVM classifier. The accuracy, precision, and specification were 94.26. The hierarchical clustering of out-set cancer genes for normal samples and stage I was represented in Supplementary Fig. S4. The classification and clustering indicate the genes’ potential in discriminating early-stage samples from normal samples.

#### Stage progression-related biomarker

We have implemented five evaluating indices to identify the most important genes involved in the progression of breast cancer, including 1) being prognostic, 2) pattern change during stages (Ascending/descending), 3) being as a hub gene, 4) being novel, and 5) having any changes in re-wiring status. Finally, we selected the lnRNA AC025034.1 (Figs. 4,5, Table 1).

**Table 1:** Novel biomarker selection

	Ascending/descending expression pattern	Novelty	Hub property	Prognostic property	Re-wiring status
**AC025034.1**	yes	yes	In stage I	yes	In 2 stages
**PCAT19**	no	yes	In stage I	yes	In 2 stages
**AL139274.2**	no	yes	In no stage	yes	In 1 stage
**SF3B3**	yes	no	In stage II	yes	In 3 stages

## Discussion

Although there are several computational methods in cancer progression studies, as well as there are different stage-related treatments for breast cancer in the clinic, patients remained at high risk of cancer development and metastasis. Therefore, it is essential to implement new strategies to detect more crucial signatures feasible in the clinic; Amongst, topological-related approaches received less attention in the field of biomarker/risk model detection, specifically, hidden dynamic regulators active in breast cancer stages.

In this study, we conducted a comprehensive assessment of differential co-expression patterns called re-wiring, across stages (Fig. 1). We introduced a new scoring method to find breast cancer-related stage-specific subnetworks involved in cancer regulatory dynamics. Moreover, the ascending/descending oncogenes involved in cancer staging were detected. Prognostic signature and their re-wiring across breast cancer stages were computationally detected and visualized; Amongst, an essential biomarker, *AC025034.1*, which is the antisense of an oncogene, *ATP2B1*, was detected. Finally, a high-performance risk model was detected using re-wired nodes (genes).

From computational and biological points of view, not only did our detected subnetworks reveal high re-wiring among stages/normal conditions, but also they represented the pivotal biological roles in cancer stages (Figs. 2,3). On the contrary, the re-wired subnetworks did not indicate statistically mean expression differences; Such results indicate the importance of the topological methods in finding cancer-related subnetworks, even though they are not differentially expressed (Fig. 2c,d).

For the HER2 subtype, we could not detect any re-wired subnetwork. We have identified several up- and down-regulated genes between HER2 positive/negative groups which are involved in shared or differentiated signaling/metabolic networks. However, we could not identify any HER2-related differential subnetwork along four stages. This may be due to the heterogeneous nature of the disease and also the sensitivity of transcriptomics data in similar phenotypes. On the other hand, this may indicate that HER2+ and – groups have similar dynamic transcriptomics patterns.

Generally, the first stage of cancer is of great interest to scientists and physicians. Of note, in previous studies, the cancer-related phenomenons, such as “*Dysregulation of ECM*” as the tumor microenvironment-related event, as well as epigenetic perturbations of “*Histone modifications*” were reported as driver events in cancer initiation, but they were not specifically studied for the first stage of breast cancer [7, 21]. These findings were in line with our specific biological pathways found for stage I, as well as Bartkova, J., et al.’s study, which demonstrated the activation of “*DNA repair pathways*” as a body barrier against genetic instability in the early stages of breast cancer [12]; Bartkova’outcome reflects the natural body response against cancer. Accordingly, the occurrence of genetics, epigenetics, and dysregulation of tumor microenvironment might suggest several biological events result in cancer progression in the first stage (Fig. 3a); Therefore, different treatment strategies, including genetic/epigenetic-related ones might be crucial to suppress cancer progression in the first stage. Similar to stage I, Stage II as an early stage is important in detection in the clinic. Currie, E., et al. discussed in their study the cancer-related traits of differentiation loss and gaining mesenchymal status, EMT, leads to an increase in the migratory potential for tumor cells to metastasize secondary sites [22]; These pathways were detected in our findings for stage II (Fig. 3b). Therefore, we concluded that such tumor arousal at the second stage is the clue of tumor efforts to survive and prepare for metastasis even in the early stages. Additionally, such findings are consistent with the hypothesis of the metastasis parallel progression model in Klein’s study [23]. Generally, in the late stages (stage III,IV), we expect cancer cells to behave more invasively. Therefore, we expect the activation of more aggressive pathways in breast cancer. The appearance of “*Filopodia*” as a protrusion in the cell membrane, which helps tumor cells move easily, and the activation of the “*SMAD signaling pathway*”, may indicate the proof of tumor preparations for the metastasis foundation in stage III (Fig. 3. c) [8, 13, 24]. Finally, the last stage, indicating the presence of the secondary tumor, was related to “Angiogenesis” and “Morphogenesis” in several studies and our study as well, which could be proof of the importance of our topological-based scoring method in detecting stage-related subnetworks [25–27]. Such invasive pathways could confirm the biological irregularity in the latter stage, in which the power of seeding and growth of disseminated tumor cells are at the highest level (Fig. 3d). Due to the heterogeneity of cancer development and parallel metastasis progression, detected pathways might be identified in other stages too; but, we reported these pathways as the core pathways for every stage of breast cancer.

We also implemented statistical tests to detect stage-associated oncogenes; In which, two groups were of more interest due to their ascending/descending dynamic pattern through cancer progression (Fig.4). Stage-associated gene signatures, such as *SF3B3* and *PGM3* indicated ascending trends across stages (Fig. 4c, g). Contrarily, genes, such as *DMBT1* and *AC025034.1* showed descending patterns (Fig. 4a,f). The ascending/descending expression trends across stages in cancer indicate potential oncogenes that dynamically affect tumor progression. Therefore, early-stage suppression/induction of such genes may be recommended to control cancer development and better treatment responses. Moreover, such stage-related patterns were not reported in previous studies, and we tried to emphasize their oncogenic function during cancer progression and their dynamic effects on patients’ survival. Among stage-associated genes, *SF3B3* is a splicing factor in the cellular transcriptional process, and its upregulation relevance to low survival of ER-positive breast cancer patients was demonstrated by Gökmen-Polar, which their study supports our results as well (Fig. 4l) [28]. As another ascending pattern gene, *PGM3* is one of the hexosamine biosynthetic pathway enzymes that reveal a critical role in tumor progression in breast cancer [29]. Although the up/down-regulation reports in breast cancer on *SF3B3* and *PGM3* corroborate our findings, there is no report concerning the ascending trend of these genes across stages.

We know, the tumor microenvironment provides a safe condition for tumor cells during cancer progression [30]. Therefore, the interplay between dysfunctional immune surveillance and tumor microenvironment may play a pivotal role in cancer development. *DMBT1*, as a tumor suppressor and archetypal link between inflammation and cancer, may provide essential clues about how innate immunity relates to regenerative processes in cancer [31]. Concordant downregulation of *DMBT1* in breast cancer supports its potential for cancer progression across stages and might be a new target for immune therapy. (Fig. 4a). Likewise, we identified *CCL22* as the highest validated prognostic signature in 12 TCGA cancers (Fig. 6b, the highest bar); Moreover, we know it acts as a chemokine contributing to the modification of tumor microenvironment and resistance to the immune system [32]. Therefore, it could be a shared therapeutic target for immune therapy in many cancers, particularly in ER-positive breast cancer.

We also investigated four dynamic networks during cancer progression (Fig. 5). The re-wiring among prognostic genes may reveal the dynamic potential hub nodes emerging across stage transitions. Such gain/loss interactions, and weak associations in Fig. 4 for stage I, stage II, stage III, and stage IV suggest the hidden perturbations in gene regulation programs leading to re-wiring. Amongst, *PCAT19*, lncRNA, is the hub node that has lost most of its interactions while transitioning the healthy state to stage I (Fig. 5a). However, this node re-wires in stage IV and gains strong positive interaction with *AL139274.2*. Meanwhile, we identified a significant downregulation of *AL139274.2* in transition normal to stage I (Fig. 4i). We know, *AL139274.2* is antisense to tumor suppressor *ZNF292*. Therefore, we concluded it might associate with the induction of *ZNF292* activity in stage I as a body barrier against cancer initiation. But, ascending expression trend of *AL139274.2* across stages indicate tumor potentials against the body (Fig. 4i). Therefore, inducing *AL139274.2* in stage I might activate tumor suppressor *ZNF292*.

We selected the LncRNA *AC025034.1* as the most important biomarker in this study due to Table 1 indices. *AC025034.1* was a loss-interaction hub node in stage I which lost its interactions in stages II and III and finally gained an interaction in stage IV with *IGDCC4* (small yellow node in Fig. 5a,d). We know it is inversely correlated (antisense) to the *ATP2B1* (*PMCA1*). In which, *ATP2B1* upregulation has been reported in tumorigenic breast cancer cell lines previously [33]. As *AC025034.1* and *ATP2B1* have a negative association, the descending mean expression pattern of AC025034.1 (Fig. 4f) may suggest the upregulation of *ATP2B1* across stages. Accordingly, the early-stage control of *AC025034.1* would also compensate for the *ATP2B1* function in cancer cells. Our findings emphasize the dual and complex role of re-wiring and gene expression changes between *AC025034.1* and *ATP2B1* across stages.

Finally, we could identify an Overall-Survival-risk model (Fig. 6a). This model may be used to predict patients’ risk scores in the early stages. Moreover, it can be employed for more precise therapeutic decisions like the Oncotype DX (21 gene recurrence model) or MammaPrint (70 gene signature test) in the clinic [3, 4], however, to apply our model in the clinic, we need more breast cancer cohorts for further validations.

## Conclusions

In summary, comprehensive studying of stage transition pathways (early-to-late) may indicate activation of stage-specific core pathways and stage-associated oncogenes during cancer progression. Using rewired-related gene signatures could be led to latent dynamic regulators which reveal dual behaviors of being a re-wired hub node and stage-associated oncogene. These results elucidate the impressive functions of such regulators from different perspectives.

## Methods

We aimed to assess the topological and expression changes of genes involved in cancer progression, simultaneously. To reach our purpose, we designed several steps. The steps were illustrated in Fig. 1.

### Samples and expression data

The RNA-seq transcriptomics and clinical metadata for ER-positive breast cancer patients and normal cases (315 samples) were prepared out of the database of The Cancer Genome Atlas (TCGA) [34], including 40 patients in stage I, 92 in stage II, 55 in stage III, 15 stage IV, and 113 samples from normal tissues surrounding the tumor site (Supplementary Table S1). We included 23 HER2 positive and 171 HER2 negative samples, as well as 180 ER-negative samples for assessment of subnetwork preservation.

### Preprocessing, normalization, and differential analysis

The preprocessing was completed in several steps. In the first step, we removed zero expression genes or genes with Not-Available (NA) values. In the second step, genes the Count Per Million values (CPM) of which were less than 0.5 were filtered out. The most variable genes remained in the last step by filtering the first quartile of the coefficient of variation. After preprocessing, the expression data were normalized to remove the technical effect by Trimmed Mean of M-values (TMM) by the EdgeR package in R [35, 36]. Finally, we implemented connectivity filtering on the adjacency matrix to extract strongly connected genes [37]. Also, the differentially expressed genes (DEGs) were computed by the EdgeR package for all stages (FDR < 0.05).

### Differential co-expression network (DCEN) reconstructions

To reconstruct the differential co-expression network for each stage, we categorize samples into two groups for each stage, according to Table S1. The adjacency matrix was calculated using the Pearson correlation coefficient. To extract highly connected subnetworks, we filter out genes featuring low connectivity score (connectivity < 0.1) according to eq. (1) [37].

1 $${k}_i={\sum}_{i\ne j}{a}_{ij}$$

where *k*_*i*_ is the connectivity score of the node *i.* Also, *a*_*ij*_ is the edge weight or the correlation coefficient of genes *i* and *j*.

Finally, the differential network for every stage was reconstructed using the DiffCoEx method [10].

### Subnetwork extraction

We clustered four DCENs, using the hybrid hierarchical clustering method. Moreover, the subnetworks were merged with heights lower than 0.2. The hybrid method is a bottom-up algorithm that can better determine far cluster members and is a mixture of Partition A round Medoids (PAM) and hierarchical clustering [38]. The extracted subnetworks included highly connected protein-coding and non-coding genes.

### Subnetwork scoring

To identify breast cancer related and stage-specific subnetworks for every stage, we calculated BreastCancerStageSpecific score (BCSS) to prioritize the identified subnetworks and select the most important subnetwork for each stage, according to eq. (2):

2 $$\mathrm{BCSS}\ {score_i}={SSscore_i}^j+{BCRscore_i}^j+{{\mathrm{BCRNRscore}}_i}^j$$

Where *i* indicates stage and *j* indicates subnetwork. The SSscore is the stage-specificity score, BCRscore is the breast cancer-relation score, and BCRNRscore is the breast cancer-related non-coding RNA score. All scores are introduced in the following sections. The subnetwork with maximum value was selected as the stage-specific breast cancer-related subnetwork.

#### Stage-specificity score (SSscore)

To score stage-specificity of all identified differential subnetworks, the topological properties of subnetworks were assessed, using two combined statistics of *Z*_*summary*_ and *Median*_*rank*_ (Supplementary Table S4) [39]. Then, a permutation test was performed to check the non-randomness of the stage-specificity results. The combined statistics include 12 statistics that assess the different aspects of similarity and dissimilarity of a subnetwork in multiple conditions. Assessment of *Z*_*summary*_ which includes connectivity and density features that show the interaction pattern of genes in subnetworks. The subnetworks with *Z*_*summary*_ < 2 are less preserved (stage-specific), If 2 < *Z*_*summary*_ < 10, subnetwork is moderately stage-specific, and if *Z*_*summary*_ > 10, the subnetwork is not stage-specific, [39]. Moreover, the higher *Median*_*rank*_ indicates higher stage-specificity. The subnetworks with low *Z*_*summary*_ and high *Median*_*rank*_ scored as high stage-specific selection for each stage, individually. The SSscore calculated according to eq. (3).

3 $${SSscore_i}^j= (1-\left(\mathit{\operatorname{rescale}}{\left({Z_{summary}}\right)}\right)+ \mathit{\operatorname{rescale}}{\left({Median_{rank}}\right)})$$

Where *i* indicates stage,  *j* indicates subnetwork. The scores were rescaled between zero and one.

#### Breast cancer-relation score (BCRscore)

DisGeNET database was downloaded, and protein-coding genes for breast cancer were queried. Fisher’s exact test was implemented for every stage subnetworks and the most breast cancer-relevant subnetworks were nominated (*P*-value< 0.05) [40, 41] (Supplementary Table S5). The breast cancer-relation scores were calculated according to eq. (4).

4 $${BCRscore_i}^j= rescale\left(- Log\left( Fishe{r}^{\prime } sExactTestP{value}_i^j\right)\right)$$

Where *i* indicates stage and *j* indicates subnetwork. The scores were rescaled between zero and one.

#### Breast cancer-relation non-coding score (BCRNCRscore)

The manual literature review strategy and Lnc2Catlas webserver were implemented to determine breast cancer non-coding RNAs and their biological functions [42]. The BRNCRscore was calculated according to eq. (5). The validation list of non-coding signatures is in supplementary Table S7.

5 $${BCRNCRscore}_i^j=\frac{Number\ of\ NC-{RNAs\ reported\ in\ breast\ cancer}_i^j}{Totall\ number\ of\ non-{coding\ RNAs\ in\ the\ subnetwork}_i^j}$$

Where *i* indicates stage and *j* indicates subnetwork.

### Gene set enrichment analysis

ClueGO plugin in Cytoscape was applied to facilitate biological interpretations of top score subnetworks and their association to common signaling pathways, biological functions, and cellular compartments (*P*-value< 0.05) [43, 44].

### Overall survival analyses, stage-rewiring network reconstruction, and trend assessment

The survival analysis was conducted on stage-specific subnetworks to detect the prognostic genes using the Log-Rank test and Kaplan-Meier curves (P-value< 0.05) [45] (Supplementary Table S7). We also performed the stepwise feature selection to detect covariates with Variance Inflating Factor (VIF) less than10. Finally, to develop an Overall-survival-risk model to stratify patients into low, meditate, and high-risk groups, the predictive cox-PH model of 12 covariates was fitted to the survival data (Supplementary Table S8,S9,S10). In this step, we fitted the model and extracted the hazard ratios (HRs). We computed the first and third quartiles of HRs. The patients with HRs less than the first quartile were categorized as the low-risk group, the patients with HR between the first and third quartiles were categorized as the medium-risk group, and finally, patients with HRs greater than the third quartile were categorized as the high-risk group. Then, the concordance index was employed to assess the Overall-survival-risk model performance. Additionally, we evaluated the normality distribution of 50 prognostic genes using the Shapiro-Wilk test, as well as Kruskal–Wallis and Post-Hock test to determine stage-associated prognostic biomarkers (Benjamini-Hochberg adjustment, P-value< 0.05). They were categorized into three groups of ascending, descending, and outset-cancer groups. The ascending/descending groups were called stage-associated biomarkers.

To identify changes in the regulation of 50 prognostic gene expression programs across stages, we reconstructed the co-expression networks for every four stages and the normal condition. And, we filter out the interaction weights using cutoff < 0.7 (significant high correlations remained). Finally, the ‘loss’, ‘gain’, and ‘reversed’ associations for edges were identified.

### Validation

The GSE3494 was normalized using the RMA method. We assessed the OS status of our prognostic genes using the median as the cutoff. The overall survival of 50 prognostic genes was also assessed by GEPIA and SurvExpress webserver for all 33 cancers in TCGA (cutoff = median) [46, 47] (Supplementary Table S7). To investigate the outset-cancer group genes in discriminating normal and stage I, the hierarchical clustering and support vector machine (SVM) classification was implemented. The five-fold cross-validation method with linear kernel was used (training percentage = 80) [48]. To assess the stage-specificity of final detected subnetworks for every stage for ER-positive patients, the Z_summery_ and Median_rank_ values were computed for every stage of ER-negative patients as well (Supplementary Table S4).

## Supplementary Information

**Additional file 1.**

## Data Availability

The datasets were analyzed during the current study are available in the TCGA repository [https://portal.gdc.cancer.gov/], and NCBI under accession number GSE3494 [https://www.ncbi.nlm.nih.gov/geo/query/acc.cgi?acc=gse3494c]. The authors affirm that all data necessary for confirming the conclusions of the article are present within the article, figures, and tables.

## Abbreviations

BCSS: BreastCancerStageSpecific score
BCRscore: Breast cancer-relation score
BCRNCRscore: Breast cancer-relation Non-coding score
EMT: Epithelial mesenchymal transition
ER +: estrogen-receptor-positive
lncRNA: Long non-coding RNA
Non-coding: NC
OS: Overall Survival
PC: Protein Coding
SSscore: Stage-specificity score
