# Supplementary material for: Re-wiring and gene expression changes of AC025034.1 and ATP2B1 play complex roles in early-to-late breast cancer progression
Source: BMC Genom Data. 2022 Jan 14;23:6. doi: 10.1186/s12863-021-01015-9 (PMC8759272; doi:10.1186/s12863-021-01015-9)
Supplement: Supplementary file 1 — Additional file 1. [file 12863_2021_1015_MOESM1_ESM.docx]

**Re-wiring and gene expression changes of AC025034.1 and ATP2B1 play complex roles in early-to-late breast cancer progression**

## **SUPPLEMENTARY METHODS**

## Differential co-expression network reconstruction

To reconstruct differential networks, we categorize samples into two groups as Table S1. The adjacency matrix was calculated using the Pearson correlation coefficient for every group.

**Table S1 Differential Network Categories**

| **Group 1** | **Group2** |
| --- | --- |
| StageI samples | StageII, III, IV Samples |
| StageII samples | StageI,III,IV Samples |
| StageIII samples | StageI,II,IV Samples |
| StageIV samples | StageI, II, III Samples |

We also checked the difference between HER2 subtypes (HER2-positive, HER2-negative), implementing t-test, PCA, and hierarchical clustering. There was no significant difference between HER2+ and HER2- subtypes. In order to implement the t-test we checked the homogeneity of variance. As p-value > 0.05, therefore two subtypes had equal variance and also equal mean (Table S2). The t-test showed no difference between HER2 subtypes (Table S3).

Table S2 homogeneity of variance

| **F p-value** | |
| --- | --- |
| **F** | p-value = 0.8117 |

Table S3 t-test

| **Two Sample t-test** | |
| --- | --- |
| t = -0.2779 | p-value = 0.7814 |

The result of PCA and hierarchical clustering on HER2 subtypes were illustrated in Figure S1 and Figure S2, respectively.


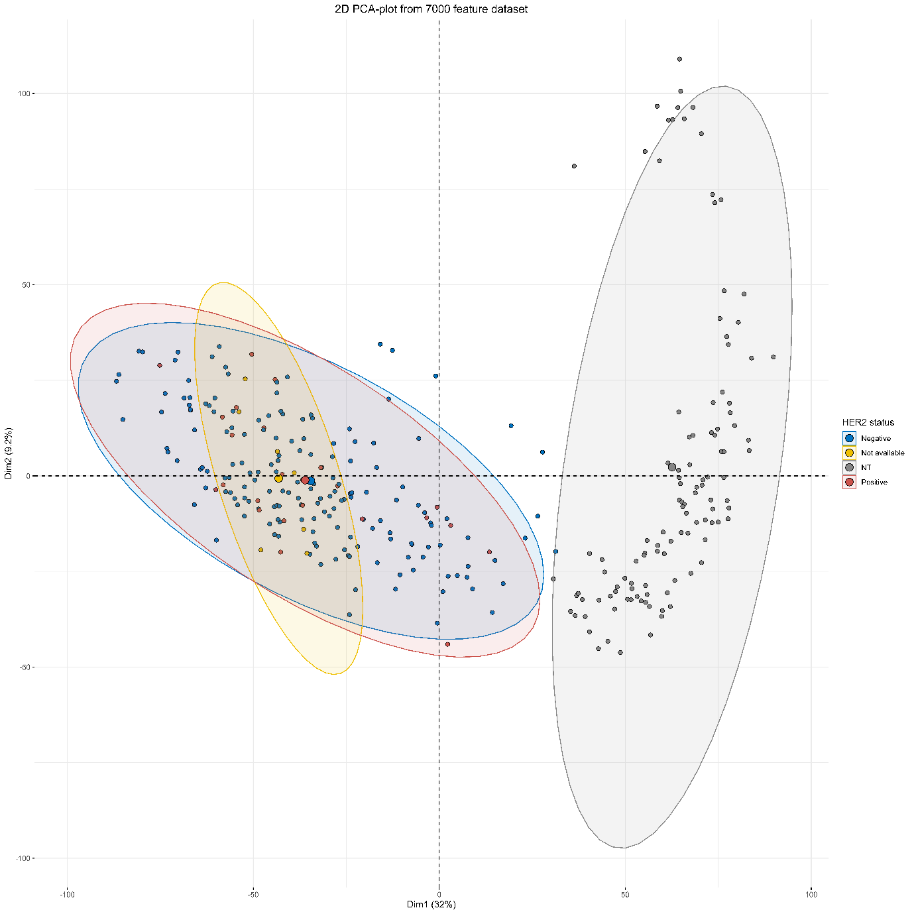


Fig. S1 the PCA result on HER2 subtypes. The red circle represents HER2+, Yellow represents unavailable sample subtype, blue represents HER2-, and the grey one reverent normal samples.


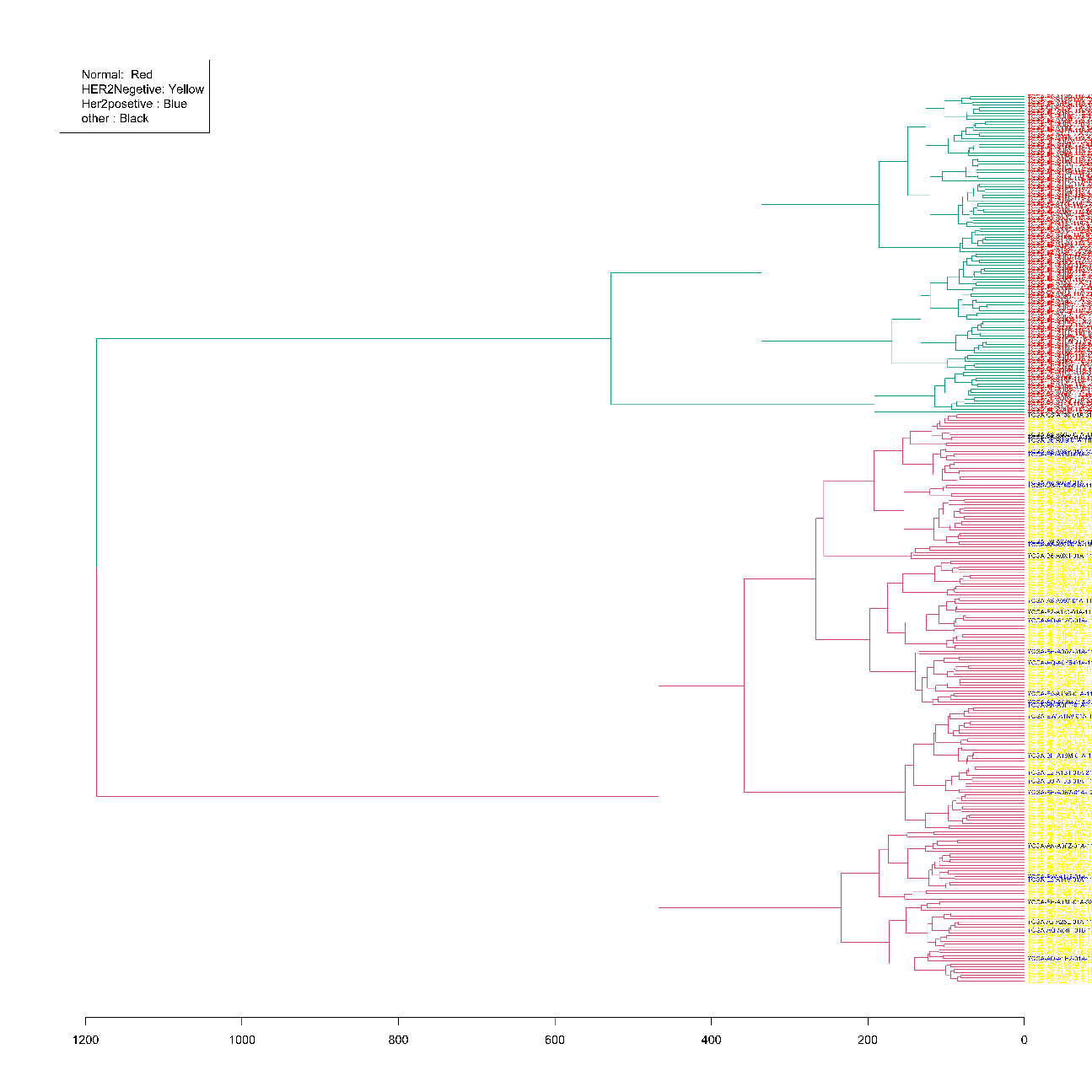


Fig. S2 hierarchical clustering on samples

## Subnetwork extraction

We clustered networks by hierarchical clustering to extract subnetworks. The clusters with a distance lower than 0.2 were merged. The extracted subnetworks were illustrated by different colors in Figure S3.


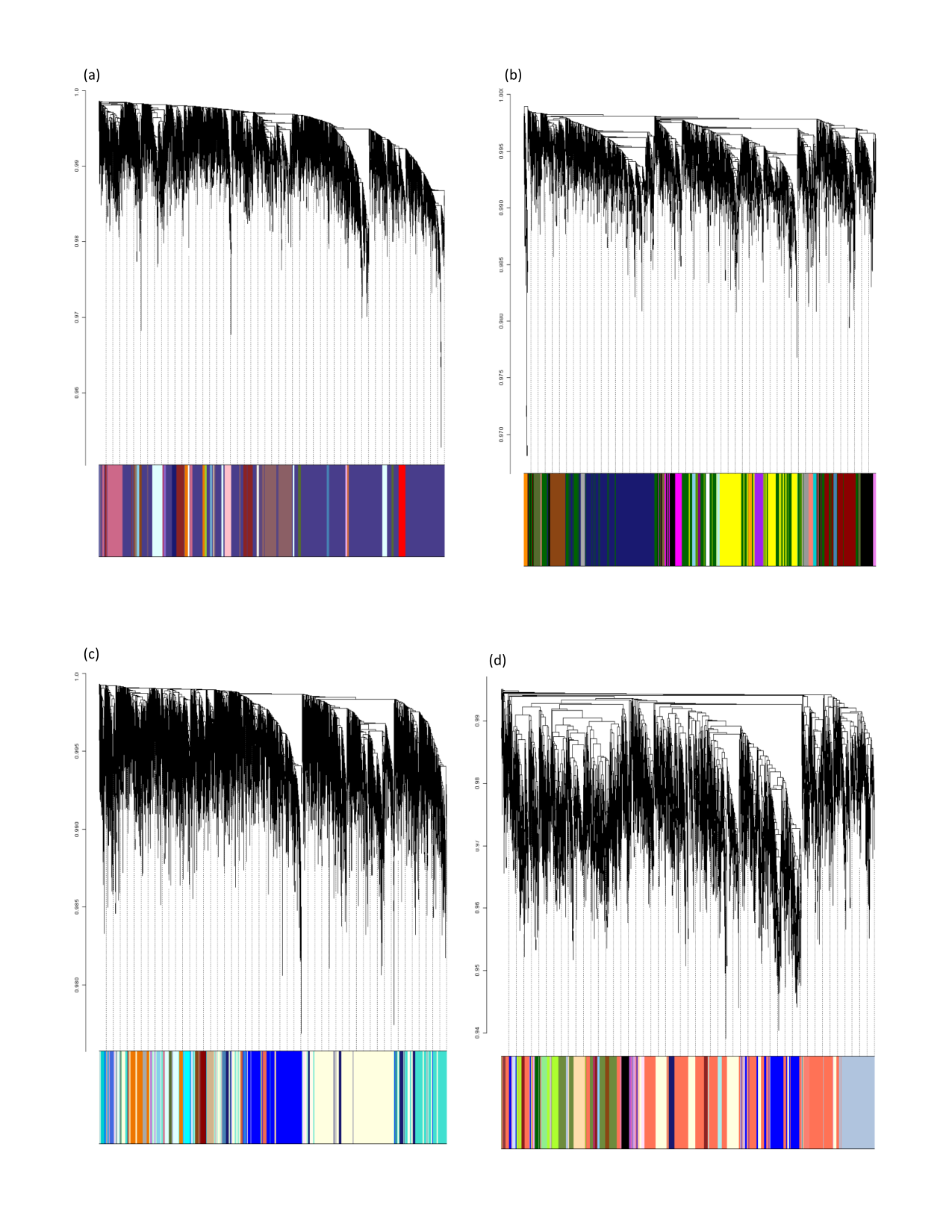


Fig. S3 Subnetwork color assignment. Each color represents a subnetwork for a) stage I, b) stage II, c) stage III, and d) stage IV.

## Stage-specific assessment of subnetworks

The formulas of combined statistics are in equations (3) and (4)

$Z_{summery}= \frac{Z_{density}+Z_{connectivity}}{2}$ (3)

${Median}_{rank}= \frac{medinRank.density+medianRank.connectivity}{2}$ (4)

The internal statistics calculated by formulas in Equation (5-8)

$Z_{connectivity}=Medin\left( Z_{cor.KIM}Z_{cor.KME}Z_{men.KME} \right)$ (5)

$Z_{density}=median\left( Z_{menCor}Z_{meanAdj}Z_{propVarWxpl}Z_{meanKME} \right)$ (6)

${medianRank.connectivity}^{\left( q \right)}= {median}_{a\in Connectivity statistics}\left( {rank}_{a}^{\left( q \right)} \right)$ (7)

${medianRank.density}^{\left( q \right)}={median}_{a\in Density statistics}\left( {rank}_{a}^{\left( q \right)} \right)$ (8)

The summery of stage-specificity statistics of $Z_{\mathrm{summery}}$ and $\mathrm{Median}_{\mathrm{rank}}$for every subnetwork were reported in Table S4. The specificity of a subnetwork is assessed by the reference stage compared to the rest of the stages and the normal condition. The results demonstrate conservation for reference condition subnetworks. Moreover , the $Z_{\mathrm{summery}}$ and $\mathrm{Median}_{\mathrm{rank}}$ were reported her for ER-negative group. ER-negative patients in every for stage of I, II, III, IV are 21, 110, 34, and 6 respectively.

Table S4 Stage-specific statistics

| Reference stage | Subnetwork  Name | Subnetwork Size | $\mathbf{Z}_{\mathbf{summery}}$ | | | |  | $\mathbf{Median}_{\mathbf{rank}}$ | | | |  |
| --- | --- | --- | --- | --- | --- | --- | --- | --- | --- | --- | --- | --- |
|  |  |  | **stageII** | **stageIII** | **stageIV** | **Normal** | **ER-** | **stageII** | **stageIII** | **stageIV** | **Normal** | **Er-** |
| I | **Bisque4** | 37 | 1.80 | 2.30 | 0.52 | 0.11 | 0.7 | 18 | 20 | 24 | 22 | 19 |
|  | **Florawhite** | 40 | -0.32 | 0.37 | 0.65 | 0.41 | -.1 | 26 | 25 | 24 | 21 | 23 |
|  | **Plum2** | 34 | 0.63 | 1.69 | 0.32 | 0.64 | -.1 | 22 | 21 | 23 | 25 | 24 |
|  | **YellowGreen** | 48 | 1.12 | 0.74 | 2.76 | 0.70 | 2 | 24 | 26 | 21 | 25 | 19 |
|  |  |  | **stageI** | **stageIII** | **stageIV** | **Normal** |  | **stageI** | **stageIII** | **stageIV** | **Normal** |  |
| II | **SkyBlue** | 33 | 1.69 | 2.22 | 1.50 | 1.44 | 2.5 | 19 | 21 | 17 | 14 | 16 |
|  | **Orange** | 38 | 2.53 | 3.40 | 1.12 | 0.62 | -1.1 | 17 | 19 | 21 | 20 | 21.5 |
|  |  |  | **stageI** | **stageIII** | **stageIV** | **Normal** |  | **stageI** | **stageIII** | **stageIV** | **Normal** |  |
| III | **FloraWhite** | 51 | 1.36 | 3.17 | 1.05 | 2.95 | -.3 | 21 | 28 | 29 | 18 | 51 |
|  | **HoneyDew1** | 37 | 1.31 | 2.25 | 0.87 | 0.15 | -1.2 | 27 | 27 | 28 | 27 | 37 |
|  | **MediumOrchid** | 30 | -0.09 | 1.57 | 1.72 | 0.73 | -0.6 | 28 | 31 | 25 | 25 | 30 |
|  | **SteelBlue** | 79 | 2.74 | 3.98 | 1.01 | 0.28 | 0.8 | 21 | 29 | 28 | 28 | 79 |
|  |  |  | **stageI** | **stageIII** | **stageIII** | **Normal** |  | **stageI** | **stageIII** | **stageIII** | **Normal** |  |
| IV | **IndianRed4** | 40 | -0.64 | 0.18 | 0.24 | 0.25 | - | 25 | 31 | 29 | 28 | - |
|  | **LightGreen** | 90 | 0.97 | 0.15 | 0.17 | 0.39 | - | 23 | 27 | 29 | 25 | - |
|  | **LightPink3** | 33 | 0.35 | 1.06 | 1.20 | 0.35 | - | 25 | 24 | 23 | 25 | - |
|  | **LightSteelblue1** | 60 | -1.26 | 0.76 | 0.88 | 0.47 | - | 30 | 30 | 30 | 26 | - |
|  | **Plum** | 43 | -0.15 | 0.03 | 0.33 | 0.75 | - | 27 | 30 | 31 | 31 | - |
|  | **Salmon2** | 36 | -0.45 | 0.55 | 0.20 | 0.23 | - | 26 | 27 | 27 | 29 | - |

*Note.*

- If $\boldsymbol{Z}_{\boldsymbol{summery}}\boldsymbol{<2,}$ then the subnetwork is stage-specific.
- if $\mathbf{2<}\boldsymbol{Z}_{\boldsymbol{summery}}$<10, then the subnetwork is moderately stage-specific.
- if $\boldsymbol{Z}_{\boldsymbol{summery}}\mathbf{>10}$, then the subnetwork is not stage-specific in reference and test conditions.
- More $\mathrm{median}_{\mathrm{Rank}}$, represents more stage-specificity.
- Due to small number of ER-negative (ER-) patients in stage IV (n=6), we could not detect the specificity statistics.

## Breast-cancer-relevant subnetwork assessment

We performed the disease-associated subnetwork assessment test in order to prioritize the biological importance of subnetworks. The BCRscores were reported in Table S5.

Table S5 Breast cancer-associated subnetworks

| **Disease** | **Stage** | **Subnetwork Name** | **Subnetwork size** | **P.C Transcripts** | **N.C Transcripts** | **N.C reported** | **Associated Transcripts to Brest Cancer** | **p-value** | **minuslogPvalue** | **BCRscore** |
| --- | --- | --- | --- | --- | --- | --- | --- | --- | --- | --- |
| **Breast Cancer** | **I** | FloraWhite | 40 | 29 | 11 | 4 | 10 | 1.30E-05* | 4.88 | 0.37 |
|  |  | Plum2 | 34 | 27 | 7 | 1 | 2 | 0.03* | 1.52 | 0.11 |
|  |  | YellowGreen | 48 | 44 | 4 | 1 | 21 | 1.30E-13* | 12.88 | 1 |
|  |  | bisque4 | 48 | 44 | 4 | 1 | 21 | 2.50E-02* | 1.60 | 0.12 |
|  | **II** | Orange | 38 | 34 | 4 | 1 | 14 | 5.20E-12* | 11.28 | 0.87 |
|  |  | SkyBlue | 33 | 25 | 8 | 1 | 7 | 2.40E-05* | 4.61 | 0.35 |
|  | **III** | FloraWhite | 51 | 35 | 16 | 4 | 12 | 1.80E-06* | 5.74 | 0.44 |
|  |  | HoneyDew1 | 37 | 11 | 26 | 2 | 2 | 0.17 | 0.76 | 0.05 |
|  |  | MediumOrchid | 30 | 15 | 15 | 2 | 5 | 2.40E-03* | 2.61 | 0.20 |
|  |  | SteelBlue | 79 | 58 | 21 | 3 | 13 | 1.10E-04* | 3.95 | 0.30 |
|  | **IV** | IndianRed4 | 40 | 20 | 20 | 3 | 6 | 3.40E-04* | 3.46 | 0.26 |
|  |  | LightGreen | 90 | 44 | 46 | 4 | 5 | 0.06 | 1.22 | 0.09 |
|  |  | LightPink3 | 33 | 15 | 18 | 4 | 2 | 0.17 | 0.76 | 0.05 |
|  |  | LightSteelBlue1 | 60 | 21 | 39 | 2 | 0 | 1 | 0 | 0 |
|  |  | MediumOrchid | 44 | 14 | 30 | 3 | 2 | 0.16 | 0.79 | 0.06 |
|  |  | Plum | 43 | 23 | 20 | 3 | 2 | 0.32 | 0.49 | 0.03 |
|  |  | Salmon2 | 36 | 17 | 19 | 1 | 0 | 1 | 0 | 0 |

Abbreviations: N.C Transcripts: None Coding Transcripts; P.C Transcript: Protein Coding Transcripts.

* P-value <0.05

## Breast cancer-related stage-specific scores

To identify breast cancer stage-specific subnetworks for every stage, we calculated the BreastCancerStageSpecific score (BCSS) to prioritize the identified subnetworks and select the most important subnetwork. BCRscore indicates breast cancer-relation scores, the SSscore indicates stage-specificity scores, BCNCRscore indicate breast cancer non-coding RNA scores, and BCSSscore indicates final score to select a subnetwork for each stage. The highest score of subnetworks for each stage indicates the most important subnetwork. All scores have been reported in Table S6.

**Table S6 Scores of subnetwork**

| **Stage** | **Subnetwork** | **BCRscore** | **SSscore** | **BCRNCRscore** | **BCSSscore** |
| --- | --- | --- | --- | --- | --- |
| **I** | FloraWhite | 0.37917392 | 2 | 0 | ***2.37917392*** |
|  | Plum2 | 0.11818035 | 0.86 | 0.142857143 | 1.121037493 |
|  | YellowGreen | 1 | 1 | 0 | 2 |
|  | bisque4 | 0.12432508 | 0.57 | 0 | 0.69432508 |
| **II** | Orange | 0.87567492 | 2 | 0 | ***2.87567492*** |
|  | SkyBlue | 0.35851067 | 1 | 0 | 1.35851067 |
| **III** | FloraWhite | 0.44580958 | 2 | 0.1875 | ***2.63330958*** |
|  | HoneyDew1 | 0.05971967 | 0.56 | 0 | 0.61971967 |
|  | MediumOrchid | 0.20330415 | 1.25 | 0 | 1.45330415 |
|  | SteelBlue | 0.30720083 | 1 | 0 | 1.30720083 |
| **IV** | IndianRed4 | 0.26916854 | 1.61 | 0 | ***1.87916854*** |
|  | LightGreen | 0.09481945 | 0.52 | 0.02173913 | 0.63655858 |
|  | LightPink3 | 0.05971967 | 0 | 0 | 0.05971967 |
|  | LightSteelBlue1 | 0 | 1.5 | 0 | 1.5 |
|  | MediumOrchid | 0.06176288 | 0.91 | 0 | 0.97176288 |
|  | Plum | 0.03840197 | 0.89 | 0 | 0.92840197 |
|  | Salmon2 | 0 | 1 | 0.052631579 | 1.052631579 |

The bold and italic numbers in the last column indicates the selected subnetwork for each stage

## Overall survival analysis

The survival analysis was performed (P-value <0.05) for stage-specific subnetworks using the *survival* package in R and identified 50 significant genes. GraphPad Prism Software windows version8 (San Diego, California USA, www.graphpad.com) for plotting the Log-Rank test and Kaplan- Meier curves [1]. The Log-Rank test results are in Table S7.

Table S7 Overall survival significant genes (coding and non-coding genes)

| Stage | Transcript Symbol | Transcript Type | Subnetwork | Expression-Survival Relation | Log-Rank | Validation | | | | |
| --- | --- | --- | --- | --- | --- | --- | --- | --- | --- | --- |
|  |  |  | Name |  | P-value | Article review | Gepia | Survexpress | R | Human Protein Atlas |
| IV | LINC01612 | lincRNA | salmon2 | High | 0.034 |  |  |  |  |  |
| IV | AC046143.1 | antisense | salmon2 | Low | 0.025 |  |  | TCGA2016 |  |  |
| IV | PTGIS | protein_coding | salmon2 | Low | 0.01 |  |  |  |  | renal, Ovarian |
| IV | C21orf62 | protein_coding | IndianRed4 | High | 0.00023 |  |  | GSE9893, GSE19536 |  | renal |
| IV | WNT2B | protein_coding | IndianRed4 | Low | 0.0082 | renal^46^ |  | GSE9893, TCGA2016 |  |  |
| IV | PGM3 | protein_coding | IndianRed4 | High | 0.049 | breast[2] | yes | GSE20685 |  | Ovarin, breast, cervical, renal |
| IV | SCRN1 | protein_coding | LightGreen | High | 0.0052 | Gastric^50^ ,breast^44^  CRC[3] | yes | TCGA2016, GSE20685 |  |  |
| IV | TIRAP | protein_coding | LightGreen | High | 0.02 | CRC[4] |  | GSE9893 |  |  |
| IV | OSTM1 | protein_coding | LightGreen | High | 0.0011 |  | yes | TCGA2016 |  |  |
| IV | CTSF | protein_coding | LightGreen | High | 0.0011 |  |  | TCGA2016 |  | renal |
| IV | AL121917.1 | antisense | LightGreen | High | 0.0089 |  |  |  |  |  |
| IV | SLC16A2 | protein_coding | LightPink3 | High | 0.026 |  | yes | GSE20685 |  | breast, renal, urothelial |
| III | AC004540.2 | antisense | FloraWhite | Low | 0.0082 |  | yes |  |  |  |
| III | NME8 | protein_coding | FloraWhite | Low | 0.012 |  |  |  |  |  |
| III | PALM | protein_coding | FloraWhite | Low | 0.015 |  |  | TCGA2016 |  | renal, pancreatic |
| III | GAPT | protein_coding | FloraWhite | Low | 0.014 | gastric[5] |  | TCGA2016, GSE20685 |  |  |
| III | ANKRD18EP | processed_pseudogene | FloraWhite | High | 0.018 |  |  |  |  |  |
| III | WFDC6 | protein_coding | SteelBlue | Low | 0.0082 |  | yes |  |  |  |
| III | ITGB5 | protein_coding | SteelBlue | Low | 0.005 | hepatocellular carcinoma^51^ |  | GSE3143 |  | pancreatic |
| III | LRRC37A11P | pseudogene | SteelBlue | Low | 0.038 |  |  |  |  |  |
| III | GPC1 | protein_coding | SteelBlue | Low | 0.038 | esophagus[6]  ,pancreatic[7] |  | TCGA2016 |  | endometrial, colorectal, liver |
| III | TPSAB1 | protein_coding | SteelBlue | Low | 0.047 |  |  | GSE20685, GSE11121 |  | head and neck |
| III | TCEAL2 | protein_coding | SteelBlue | Low | 0.0013 |  |  | GSE20685 |  |  |
| III | DMBT1 | protein_coding | SteelBlue | Low | 0.041 | breast[8] |  | GSE3143 |  | endometrial |
| III | AL139274.2 | antisense | HoneyDew1 | High | 0.0082 |  |  |  |  |  |
| III | AL845472.2 | TEC | HoneyDew1 | High | 0.0011 |  |  |  |  |  |
| III | AC025034.1 | antisense | HoneyDew1 | Low | 0.04 |  |  |  |  |  |
| II | DGKG | protein_coding | orange | High | 0.027 |  |  | GSE9893, GSE1379 |  |  |
| II | CCL22 | protein_coding | orange | Low | 0.0051 |  |  | GSE9893, TCGa2016 |  | colorectal, head and neck, endometrial |
| II | TNN | protein_coding | SkyBlue | Low | 0.0033 |  | yes | GSE20985 |  |  |
| II | IGDCC4 | protein_coding | SkyBlue | Low | 0.048 |  |  |  |  |  |
| I | ACTN2 | protein_coding | FloraWhite | High | 0.043 | AML[9] |  | GSE9893, GSE11121 |  |  |
| I | TMEM156 | protein_coding | FloraWhite | Low | 0.0013 |  | yes | TCGA2016, GSE20685 |  | breast |
| I | ROPN1B | protein_coding | FloraWhite | Low | 0.014 | Breast [10] in cox | yes |  |  |  |
| I | DACT2 | protein_coding | Plum2 | Low | 0.013 |  | yes | TCGA2016 |  | renal, endometrial |
| I | GSG1L | protein_coding | Plum2 | Low | 0.018 |  |  | GSE20685 |  |  |
| I | CASC9 | lincRNA | Plum2 | High | 0.03 | Cervical ^52^, esaphagus[11]  ,breast^53^ |  |  | GSE3494 |  |
| I | FRMPD1 | protein_coding | Bisque4 | High | 0.041 |  |  | TCGA2016 |  |  |
| I | CAVIN4 | protein_coding | Bisque4 | High | 0.038 |  | yes | GSE20685 |  |  |
| I | SF3B3 | protein_coding | Bisque4 | High | 0.0037 | ER+ breast^54^ | yes | GSE19536 |  |  |
| I | PCDHGA1 | protein_coding | Bisque4 | High | 0.043 |  | yes | GSE9893, GSE11121 |  |  |
| I | ADGRG1 | protein_coding | Bisque4 | High | 0.017 | breast[12] |  |  |  | renal, head and neck |
| I | AC092142.1 | antisense | Bisque4 | High | 0.023 |  |  |  |  |  |
| I | AC005618.1 | lincRNA | Bisque4 |  | 0.015 |  |  |  |  |  |
| I | AC008969.1 | processed_transcript | Bisque4 | Low | 0.04 |  |  |  |  |  |
| I | PDLIM4 | protein_coding | YellowGreen | Low | 0.039 |  |  |  |  |  |
|  |  |  |  |  |  |  |  |  |  |  |
| I | SRARP | protein_coding | YellowGreen | Low | 0.008 |  |  |  |  |  |
| I | APOBEC3D | protein_coding | YellowGreen | Low | 0.0044 |  |  |  |  | Renal, breast, cervical, urothelial |
| I | PCAT19 | lincRNA | YellowGreen | Low | 0.022 | prostate[13] |  |  | GSE3494 |  |
| I | SEMA3G | protein_coding | YellowGreen | Low | 0.016 | breast[14] |  |  |  | Renal |

*Note*: All the results are at 0.05 significant level.

“High” on column five means High expression of selected transcript relates to the low survival of patients.

“Low” on column five means Low expression of selected transcript relates to the low survival of patients.

## Cox PH results

The cox-PH results were implemented to stratify patients into low, medium, and high-risk groups. The step-wise cox-PH was implemented to select covariates with suitable VIF (Variance inflation factor) value (VIF < 10). The selected covariates and their hazard ratio and p-values are in Table S8. The model fitness results are in Table S9. The likelihood ratio test, Wald test, and score test all were significant. The concordance index (C-Index) evaluates the model discrimination in survival analysis[15] and it is 0.89 in our result, which implies a good model fitness.

Table S8 the VIF Cox-PH covariates result

| Covariates | Hazard Ratio | P-value |
| --- | --- | --- |
| AC004540.2 | 2.26457203 | 0.00293184 ** |
| [GPC1](http://asia.ensembl.org/homo_sapiens/Gene/Summary?db=core;g=ENSG00000063660) | -1.87453669 | 0.00136171 ** |
| [ACTN2](http://asia.ensembl.org/homo_sapiens/Gene/Summary?db=core;g=ENSG00000077522) | 1.9768435 | 0.000013168 *** |
| [LINC01612](http://asia.ensembl.org/homo_sapiens/Gene/Summary?db=core;g=ENSG00000250266) | 0.94874003 | 0.00702618 ** |
| [LRRC37A11P](http://asia.ensembl.org/homo_sapiens/Gene/Summary?db=core;g=ENSG00000214553) | 1.86424173 | 0.00757313 ** |
| [SRARP](http://asia.ensembl.org/homo_sapiens/Gene/Summary?db=core;g=ENSG00000183888) | 0.69623386 | 0.01307738 * |
| [ADGRG1](http://asia.ensembl.org/homo_sapiens/Gene/Summary?db=core;g=ENSG00000205336) | 0.17556675 | 0.00103306 ** |
| [PCAT19](http://asia.ensembl.org/homo_sapiens/Gene/Summary?db=core;g=ENSG00000267107) | 0.07981218 | 0.00032473 *** |
| [ITGB5](http://asia.ensembl.org/homo_sapiens/Gene/Summary?db=core;g=ENSG00000082781) | 0.16599296 | 0.00252455 ** |
| [GPC1](http://asia.ensembl.org/homo_sapiens/Gene/Summary?db=core;g=ENSG00000063660) | 4.64876918 | 0.02202285 * |
| [SEMA3G](http://asia.ensembl.org/homo_sapiens/Gene/Summary?db=core;g=ENSG00000010319) | 4.57177742 | 0.00157689 ** |
| [SF3B3](http://asia.ensembl.org/homo_sapiens/Gene/Summary?db=core;g=ENSG00000189091) | 6.78540109 | 0.00723171 ** |

* P-value <0.05

** P-value <0.01

*** P-value <0.001

Table S9 cox PH model fitness

| Test Type |  | P-value |
| --- | --- | --- |
| Concordance | 0.89 |  |
| Likelihood ratio test |  | 3.764E-07*** |
| Wald test |  | 0.00008676*** |
| Score (log-rank) test |  | 0.000001462*** |

*** P-value <0.001

The cox-PH is the proportional hazard assumption is one of the essential assumptions. We implemented the proportional hazard assumption and all the covariates and the global option of the test accepted the null assumption, therefor the proportional hazard assumptions were correct (Table S10).

Table S10 PH assumption for Cox model

| covariate |  | P-value |
| --- | --- | --- |
| AC004540.2 |  | 0.67262633 |
| [GPC1](http://asia.ensembl.org/homo_sapiens/Gene/Summary?db=core;g=ENSG00000063660) |  | 0.63323588 |
| [ACTN2](http://asia.ensembl.org/homo_sapiens/Gene/Summary?db=core;g=ENSG00000077522) |  | 0.37595866 |
| [LINC01612](http://asia.ensembl.org/homo_sapiens/Gene/Summary?db=core;g=ENSG00000250266) |  | 0.63412608 |
| [LRRC37A11P](http://asia.ensembl.org/homo_sapiens/Gene/Summary?db=core;g=ENSG00000214553) |  | 0.25666198 |
| [SRARP](http://asia.ensembl.org/homo_sapiens/Gene/Summary?db=core;g=ENSG00000183888) |  | 0.98078221 |
| [ADGRG1](http://asia.ensembl.org/homo_sapiens/Gene/Summary?db=core;g=ENSG00000205336) |  | 0.28297516 |
| [PCAT19](http://asia.ensembl.org/homo_sapiens/Gene/Summary?db=core;g=ENSG00000267107) |  | 0.21130584 |
| [ITGB5](http://asia.ensembl.org/homo_sapiens/Gene/Summary?db=core;g=ENSG00000082781) |  | 0.02236776 |
| [GPC1](http://asia.ensembl.org/homo_sapiens/Gene/Summary?db=core;g=ENSG00000063660) |  | 0.19662465 |
| [SEMA3G](http://asia.ensembl.org/homo_sapiens/Gene/Summary?db=core;g=ENSG00000010319) |  | 0.17333216 |
| [SF3B3](http://asia.ensembl.org/homo_sapiens/Gene/Summary?db=core;g=ENSG00000189091) |  | 0.78481174 |
| GLOBAL |  | 0.34274847 |

## The assessment of prognostic genes in 33 TCGA cancer types

In the next step, we investigated the involvement of the 50 prognostic genes in all other cancers (33 cancers) using the Gepia web server [16]. The results reported in Table S11. In which, column two indicates the significant 50-prognostics genes in other cancers and Column four indicates the number of significant transcripts in every cancer.

Table S11 All-cancer prognostic value

| **Transcript name** | **Number of significant cancers** | **Cancer abbreviation** | **Number of significant transcripts** |
| --- | --- | --- | --- |
| CCL22 | 12 | KIRC | 23 |
| APOBEC3D | 9 | LGG | 18 |
| AC025034.1 | 9 | SKCM | 14 |
| PGM3 | 8 | BRCA | 13 |
| ITGB5 | 8 | HNSC | 13 |
| GPC1 | 8 | BLCA | 12 |
| CAVIN4 | 8 | MESO | 12 |
| SF3B3 | 7 | SARC | 12 |
| OSTM1 | 7 | ACC | 11 |
| TMEM156 | 6 | PAAD | 10 |
| PALM | 6 | UVM | 10 |
| GAPT | 6 | LAML | 8 |
| DMBT1 | 6 | LIHC | 8 |
| AL845472.2 | 6 | COAD | 7 |
| ACTN2 | 6 | KICH | 6 |
| TPSAB1 | 5 | KIRP | 6 |
| TNN | 5 | LUAD | 6 |
| TCEAL2 | 5 | STAD | 5 |
| SLC16A2 | 5 | THCA | 5 |
| PTGIS | 5 | READ | 4 |
| IGDCC4 | 5 | THYM | 4 |
| ADGRG1 | 5 | UCEC | 4 |
| AC046143.1 | 5 | CESC | 3 |
| TIRAP | 4 | GBM | 3 |
| SEMA3G | 4 | OV | 3 |
| SCRN1 | 4 | LUSC | 2 |
| ROPN1B | 4 | PCPG | 2 |
| PDLIM4 | 4 | CHOL | 1 |
| FRMPD1 | 4 | UCS | 1 |
| DACT2 | 4 | DLBC | 0 |
| C21orf62 | 4 | ESCA | 0 |
| ANKRD18EP | 4 | PRAD | 0 |
| AL139274.2 | 4 | TGCT | 0 |
| NME8 | 3 |  |  |
| LRRC37A11P | 3 |  |  |
| DGKG | 3 |  |  |
| CTSF | 3 |  |  |
| CASC9 | 3 |  |  |
| AC005618.1 | 3 |  |  |
| AC004540.2 | 3 |  |  |
| WNT2B | 2 |  |  |
| SRARP | 2 |  |  |
| PCDHGA1 | 2 |  |  |
| PCAT19 | 2 |  |  |
| GSG1L | 2 |  |  |
| AL121917.1 | 2 |  |  |
| WFDC6 | 1 |  |  |
| LINC01612 | 0 |  |  |
| AC092142.1 | 0 |  |  |
| AC008969.1 | 0 |  |  |

The validation figure of clustering was illustrated in figure S4. In figure S4. a, the selected oncogenes were used to cluster stages, and in part b, the clustering of patients of stage I and normal individuals were shown.


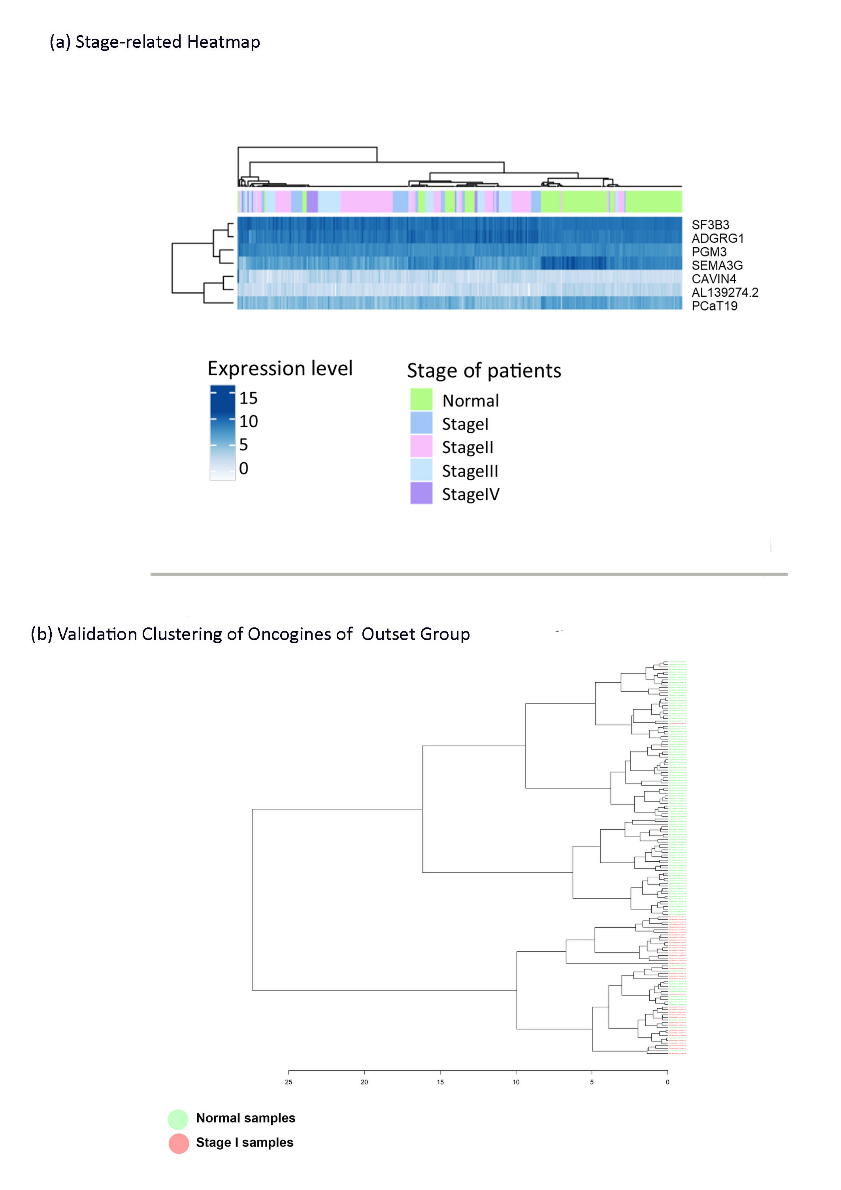


Fig. S4 Validation

# References

1. Therneau, T.J.R.S., *A Package for Survival Analysis in S. version 2.38. 2015.* 2017.

2. Ricciardiello, F., et al., *Inhibition of the Hexosamine Biosynthetic Pathway by targeting PGM3 causes breast cancer growth arrest and apoptosis.* 2018. **9**(3): p. 377.

3. Miyoshi, N., et al., *SCRN1 is a novel marker for prognosis in colorectal cancer.* 2010. **101**(2): p. 156-159.

4. Klimosch, S.N., et al., *Functional TLR5 genetic variants affect human colorectal cancer survival.* 2013. **73**(24): p. 7232-7242.

5. Song, Z., et al., *Elementary screening of lymph node metastatic-related genes in gastric cancer based on the co-expression network of messenger RNA, microRNA and long non-coding RNA.* 2018. **51**(4).

6. Nishigaki, T., et al., *Glypican-1 is a potential marker of prognosis and involved in chemoresistance of cisplatin in esophageal squamous cell cancer*. 2016, AACR.

7. Qian, J.Y., et al., *Prognostic value of glypican‑1 for patients with advanced pancreatic cancer following regional intra‑arterial chemotherapy.* 2018. **16**(1): p. 1253-1258.

8. Braidotti, P., et al., *DMBT1 expression is down-regulated in breast cancer.* 2004. **4**(1): p. 46.

9. Yang, X., et al., *High Expression Levels of ACTN1 and ACTN3 Indicate Unfavorable Prognosis in Acute Myeloid Leukemia.* 2019. **10**(18): p. 4286.

10. Su, J., et al., *Development of prognostic signature and nomogram for patients with breast cancer.* 2019. **98**(11).

11. Liang, Y., et al., *LncRNA CASC9 promotes esophageal squamous cell carcinoma metastasis through upregulating LAMC2 expression by interacting with the CREB-binding protein.* Cell Death & Differentiation, 2018. **25**(11): p. 1980-1995.

12. Ke, N., et al., *Orphan G protein–coupled receptor GPR56 plays a role in cell transformation and tumorigenesis involving the cell adhesion pathway.* 2007. **6**(6): p. 1840-1850.

13. Hua, J.T., et al., *Risk SNP-mediated promoter-enhancer switching drives prostate cancer through lncRNA PCAT19.* 2018. **174**(3): p. 564-575. e18.

14. Kotepui, M., et al., *Quantitative Real-Time RT-PCR of ITGA7, SVEP1, TNS1, LPHN3, SEMA3G, KLB and MMP13 mRNA Expression in Breast Cancer.* 2012. **13**(11): p. 5879-5882.

15. Steck, H., et al. *On ranking in survival analysis: Bounds on the concordance index*. in *Advances in neural information processing systems*. 2008.

16. Tang, Z., et al., *GEPIA: a web server for cancer and normal gene expression profiling and interactive analyses.* 2017. **45**(W1): p. W98-W102.
